# Effect of Ethanol on Parthenogenetic Activation and α-Tocopherol Supplementation during *In Vitro* Maturation on Developmental Competence of Summer-Collected Bovine Oocytes

**DOI:** 10.3390/cimb43030158

**Published:** 2021-12-16

**Authors:** Francisco Báez, Belén Gómez, Victoria de Brun, Nélida Rodríguez-Osorio, Carolina Viñoles

**Affiliations:** 1 Instituto Superior de la Carne, Centro Universitario Regional Noreste, Universidad de la República, Ruta 5, km 386, Tacuarembó 45000, Uruguay; belugoma@gmail.com; 2 Laboratorio de Endocrinología y Metabolismo Animal, Universidad de la República, Laspalces 1620, Montevideo 45000, Uruguay; videbrun@gmail.com; 3 Unidad de Genómica y Bioinformática, Departamento de Ciencias Biológicas, Centro Universitario Regional Litoral Norte, Universidad de la República, Rivera 1350, Salto 50000, Uruguay; nelida.rodriguez@unorte.edu.uy; 4 Centro de Salud Reproductiva de Rumiantes en Sistemas Agroforestales, Centro Universitario Regional Noreste, Universidad de la República, Ruta 26, km 408, Cerro Largo 37000, Uruguay; carolinavinolesgil@gmail.com

**Keywords:** antioxidant, quality blastocysts, summer, gene expression

## Abstract

The use of α-tocopherol during *in vitro* maturation (IVM) is an alternative to minimize the adverse effects of heat stress on oocyte competence. However, α-tocopherol is diluted in ethanol, which can induce oocyte parthenogenetic activation (PA). This study aimed to evaluate the role of ethanol concentration on PA and the effect of α-tocopherol supplementation during IVM on the developmental competence and the expression of key genes in blastocysts derived from summer-collected oocytes. All *in vitro* embryo production was conducted at 5% O_2_, 5% CO_2_ at 38.5 °C. Experiment 1: oocytes were cultured with or without 0.05% ethanol. As positive PA control matured oocytes were subjected to 3% or 7% ethanol for 7 min. Oocytes from all groups were placed in fertilization medium (22 h) and culture medium (9 days). Ethanol at 0.05% during IVM did not induce oocyte PA, however, 3% and 7% ethanol were effective parthenogenetic inductors. Experiment 2: oocytes were cultured in maturation medium supplemented with 0, 50, 100 and 200 μM α-tocopherol, diluted in 0.05% ethanol. After *in vitro* fertilization and embryo culture, we assessed blastocyst apoptotic index and the transcription of a panel of genes. The results showed that supplementation with 100 μM α-tocopherol reduced apoptotic index and increased the expression of *SOD2*. In conclusion, 100 μM α-tocopherol, diluted in 0.05% ethanol, can be used during IVM to embryonic quality.

## 1. Introduction

During summer, in temperate regions, grazing beef cows are exposed to acute heat stress [1], which can result in multiple physiological and cellular changes that reduce reproductive performance [2,3]. Heat stress can compromise fertility in cattle by inducing alterations in the steroidogenic capacity of the follicles. It can also cause ovulation defects, affecting oocyte competence and preimplantation embryonic development [4]. Despite the multiple detrimental effects of heat stress on the hypothalamus–pituitary–ovarian axis [5,6,7], there is increasing *in vivo* and *in vitro* evidence that oocytes are also significant targets of maternal heat stress [8]. Thus, several studies have confirmed that cumulus oocyte complexes (COCs) collected during summer show lower quality [3,9,10,11] and compromised developmental competence after *in vitro* fertilization, suggesting that summer heat stress may potentially impair oocyte quality [12,13].

One of the mechanisms by which heat stress appears to induce damage to the oocyte and developing embryo is an increase in the production of reactive oxygen species (ROS) [14]. Although ROS can regulate cell function and activate key signaling pathways [15], in excess they are also implicated in many types of cell injuries such as membrane lipid peroxidation, oxidation of nucleic acids, apoptosis and necrosis [16]. One approaches to combat the effect of heat stress on bovine oocyte developmental competence is adding thermoprotective or antioxidant molecules during *in vitro* maturation (IVM). Vitamin E or α-tocopherol is a natural antioxidant that provides cell defense against oxidative stress [17]. Previous studies reported an increase in blastocyst yield in sheep [18], pig [16], and cattle [19] with the addition of this molecule during *in vitro* culture (IVC). Supplementation of IVM medium with α-tocopherol reduced apoptotic rate in rabbit [20] and porcine [21] cumulus cells and improved oocyte developmental competence. However, the expression of genes involved in the processes of heat and oxidative stress response *(SOD2, CAT* and *HSPA1A*), pro- and anti-apoptotic activity (*BAX* and *BCL2),* and maternal recognition of pregnancy (*IFNT2)* has not been described in α-tocopherol-treated embryos, and its evaluation can provide insights into its mechanism of action [22].

Due to its hydrophobic nature, α-tocopherol is first diluted in absolute ethanol, to obtain a final 25–400 μM α-tocopherol concentration and 0.05% (*v*/*v*) ethanol in culture media. Ethanol is a known parthenogenesis activator at concentrations above 3% [23], and when associated with other molecules is used in long protocols to produce parthenogenetic embryos [24]. Although 1% ethanol and 20% O_2_ have been found to be inefficient in promoting parthenogenetic activation in bovine oocytes [23], a recent study reported that adding 0.3% ethanol to the IVM medium increased blastocyst rate, regardless of culture conditions [25]. Other studies have tested ethanol as the vehicle for the dilution of different molecules, during oocyte culture [14], but few systematic studies have investigated the role of ethanol at such low concentrations on parthenogenetic activation and embryonic development. Therefore, it is necessary to evaluate if, under physiological oxygen tension, 0.05% (*v*/*v*) ethanol can induce parthenogenetic activation in immature oocytes, in comparation with a high concentration of ethanol for a few minutes as a positive control, to promote parthenogenetic development in oocytes.

Although the beneficial effect of α-tocopherol in mammalian oocytes has been investigated, to date there are no studies on the effect of α-tocopherol (and 0.05% ethanol) supplementation during IVM and their influence on the competence of bovine oocytes collected during summer. We hypothesized that during IVM the supplementation with ethanol at 0.05% (*v*/*v*) does not promote parthenogenetic activation, but ethanol at 3 and 7% for 7 min is an effective inductor of parthenogenesis. Additionally, ethanol-diluted α-tocopherol improves the yield and quality of bovine embryos, which is reflected in the expression of target genes. The objectives of this study were to (1) measure the role of ethanol on parthenogenic activation during IVM of bovine oocytes collected during summer, and to (2) determine how α-tocopherol supplementation during *in vitro* maturation of oocytes, collected during summer, affects their nuclear maturation, fertilization rate, and the subsequent development and the expression of *SOD2, CAT*, *HSPA1A*, *BAX*, *BCL2* and *IFNT2* in the resulting embryos.

## 2. Materials and Methods

Reagents: all chemicals were purchased from Sigma-Aldrich (St. Louis, MO, USA), unless otherwise indicated.

### 2.1. Sample Collection

In this study, experiments were performed using bovine oocytes aspirated from ovaries obtained from a local slaughterhouse during summer in the southern hemisphere (between 21 December 2020 to 9 March 2021). Maximal and minimal temperatures and relative humidity were obtained from two meteorological stations of the National Research Institute for Agriculture (INIA-Uruguay), Agroclimatic Bank located in the north of the country: Salto (31°16′22′′ S, 57°53′27′′ W) and Tacuarembó (31°42′32′′ S, 55°49′36′′ W). The temperature–humidity index (THI) was calculated using the following equation:

THI=(0.8×T+(RH(%)/100)(T−14.4)+46.4)

where *T* is air temperature in degrees Celsius and *RH* is relative humidity [26].

Pools of 35–45 ovaries were transported to the laboratory in a thermal container with 0.9% NaCl (*w*/*v*) at 37 °C within 45 min of dissection. Cumulus-oocyte complexes (COCs) were aspirated from 2 to 8 mm-diameter follicles. For each repeat, around of 150 COCs with homogeneous cytoplasm and compact layers of cumulus cells were selected. All phases of *in vitro* embryo production were conducted at low oxygen tension (5% O_2_), a concentration closer to the oxygen content in the follicle and female reproductive tract [27].

### 2.2. Experimental Design

#### 2.2.1. Experiment 1

For evaluation of parthenogenetic activation of bovine oocytes in the presence of ethanol, a total of 826 COCs were used in six independent repeats. Selected COCs were maturated *in vitro* with ethanol at 0.05% (*v*/*v*) (*n* = 242) and without ethanol (*n* = 584) for 24 h. After *in vitro* maturation (IVM), 50–51 COCs per group were randomly denuded of cumulus cells, fixed, and processed for Hoechst DNA staining to determine meiotic progression. Matured COCs in the absence of alcohol were distributed into the control group (*n* = 180) and two groups that were activated with 3% (*n* = 176) and 7% (*n* = 178) ethanol (*v*/*v*) in holding media solution at 38 °C for 7 min. All groups were washed twice in holding media and cultured for 22 h in IVF-TL in absence of spermatozoa. After this period, a total of 48–53 oocytes per group were fixed and stained to determine pronuclei formation. The remaining oocytes (125–138 per group) were cultured *in vitro* for 9 days. Cleavage and blastocyst rates were determined on the third, seventh and ninth culture days, respectively. At the end of culture, embryos with a visible blastocoel were considered to be blastocysts. Parthenogenetic blastocysts of all groups (2–9 per treatments) were fixed and stained, and nuclei numbers were counted.

#### 2.2.2. Experiment 2

To examine the effects of α-tocopherol during IVM on the developmental capacity of bovine COCs, a total of 1818 COCs were used in eight independents repeats, divided into five treatments, and placed in maturation medium supplemented with different concentrations (0, 0.05% ethanol, 50, 100 and 200 µM) of α-tocopherol (*v*/*v*) for 24 h at 38.5 °C and 5% CO_2_, 5% O_2_ and 90% N_2_. After this period, a group of oocytes (*n* = 59–73 per treatment) were denuded of cumulus cells and stained to evaluate meiotic progression. Then, 1482 COCs were fertilized *in vitro*. From those, 353 presumptive zygotes (*n* = 66–73 per treatment) were denuded, fixed, and stained to determine fertilization rate. The remaining presumptive zygotes (*n* = 208–244 per treatment) were cultured *in vitro* and embryo development was assessed at day 3 (% cleavage) and day 9 (% early, expanded, and hatched blastocysts). On day 9, 10 expanded blastocysts per treatment were used for measuring the relative expression of genes involved in oxidative stress (*CAT* and *SOD2*), heat shock (*HSPA1A*), maternal recognition of pregnancy (*IFNT2*) and apoptosis (*BAX* and *BCL2*). Finally, expanded (11–14 per treatment) blastocysts were used for total cell number and apoptotic index evaluation through Terminal deoxynucleotidyl transferase mediated dUTP nick end labeling (TUNEL).

### 2.3. In Vitro Maturation

Groups of 45–50 COCs were matured *in vitro* in 500 μL medium covered with mineral oil during 24 h at 38.5 °C and 5% CO_2_, 5% O_2_ and 90% N_2_ with maximum humidity. IVM medium consisted of TCM 199 (Gibco, Grand Island, NY, USA) supplemented with 1 μg/mL Folltropin-V^®^ (Bioniche, Belleville, ON, Canada) (*v*/*v*), 5 UI/mL equine chorionic gonadotropin (Biogón^®^ Plus, Biogénesis Bagó, Provincia de Buenos Aires, Argentina) (*v*/*v*), 10% fetal bovine serum (Gibco) (*v*/*v*), 5 μg/mL gentamicin (*v*/*v*) and 0.2 mM sodium pyruvate (*w*/*v*). For α-tocopherol treatment groups, IVM medium was supplemented with different α-tocopherol (Sigma, 258024) concentrations (50, 100 and 200 μM). Antioxidant was first dissolved in absolute ethanol to obtain the stock solution of 0.1, 0.2 and 0.4 M concentration, stored in the dark at 4 °C, and diluted in maturation media to a final concentration of 50, 100 and 200 µM α-tocopherol, respectively, in 0.05% (*v*/*v*) ethanol, and prepared 4 h before culture.

### 2.4. Parthenogenetic Activation

After IVM for 24 h, COCs were washed three times in holding medium (114 mM sodium chloride, 3.2 mM potassium chloride, 0.34 mM sodium biphosphate, 0.5 mM magnesium chloride, 2.0 mM calcium chloride, 2 mM sodium bicarbonate, 10 mM sodium lactate, 0.2 mM sodium pyruvate, 4 mg/mL BSA fraction V, and 50 µg/mL gentamicin) and then incubated in 3 and 7% ethanol (*v*/*v*) in holding medium at 38 °C for 7 min. After activation, oocytes were washed three time with the same medium and transferred to 500 µL IVF-TL in absence of spermatozoa. Control group was cultured in IVF-TL in the same culture conditions, but in absence of ethanol.

### 2.5. Assessment of Meiotic Progression

Briefly, IVM COCs were denuded of cumulus cells by pipetting in phosphate-buffered saline (PBS) supplemented with 200 μg/mL hyaluronidase for 5 min. Denuded oocytes were then fixed in 2.5% paraformaldehyde (*v*/*v*) in modified phosphate-buffered saline (PBS) for 25 min and stained with 1 μg/mL Hoechst 33,342 for 5 min. These oocytes were placed on a slide and immediately observed under a fluorescence microscope Nikon Eclipse 50i equipped with a UV excitation filter at 330–385 nm and an emission filter at 420 nm. Oocytes were classified as mature (oocytes in metaphase II + polar body), immature (germinal vesicle, metaphase I, anaphase I-telophase I), and degenerated (diffuse or degraded chromatin) [28].

### 2.6. In Vitro Fertilization

Matured COCs were washed twice in *in vitro* fertilization medium (TL-IVF) containing 10 µg/mL heparin (*v*/*v*), 10 µM hypotaurine (*v*/*v*) and placed in 4-well culture dishes. Sperm cells were selected with BoviPure density gradient (Nidacon International AB, Mölndal, Sweden) by centrifugation at 300× *g* during 10 min. IVF was performed by incubating COCs with 1 × 10^6^ spermatozoa/mL in 500 μL TL-IVF medium for 22 h at 38.5 °C and 5% CO_2_, 5% O_2_, 90% N_2_ with maximum humidity.

### 2.7. Assessment of Pronucleus Formation

After 22 h in IVF-TL, COCs were denuded of cumulus cells, fixed, stained, and evaluated as in previous sections. In experiment 1, oocytes were classified according to the stage: one or two pronuclei (PN), telophase II, metaphase II or degenerated; while in experiment 2, presumptive zygotes with two PN were considered as having normal fertilization, those with three PN as polyspermic, those with one PN as asynchronous, and those without PN as unfertilized. Total fertilization rate was considered when the oocyte had one, two or more PN.

### 2.8. In Vitro Culture

At 22 h of coculture, COCs and presumptive zygotes were totally denuded of cumulus cells by pipetting in holding medium and washed twice in modified culture synthetic oviduct fluid (mSOF) [29] containing amino acids, citrate, myoinositol, 5 g/L (*w*/*v*) BSA and 0.5% FCS (*v*/*v*). Presumptive zygotes were placed in groups between 20 to 25 in mSOF and overlaid with mineral oil at 38.5 °C and 5% CO_2_, 5% O_2_, 90% N_2_ with maximum humidity for 9 days. Culture media were replaced with fresh medium (replacement 50%) at 72, 120 and 168 h of culture. Cleavage, blastocyst rate, and total blastocyst rate was measured on day 3, 7 and 9 of culture, respectively.

### 2.9. Cell Counting in Parthenogenetic Blastocysts

Blastocysts on day 9 were fixed and stained with 1 µg/mL Hoechst 33342 at 38.5 °C for 10 min, washed twice in PBS and mounted on slides. They were observed under a fluorescent microscope, and the total number of nuclei for every blastocyst was counted. Stained blastocysts were photographed, and images were evaluated using ImageJ software.

### 2.10. Assessment of Total Cell Number and DNA Fragmentation

TUNEL assay was performed as previously described [8]. Hatched and expanded blastocysts were fixed with 2.5% paraformaldehyde in PBS for 1 h at room temperature, washed in PBS and permeabilized in 0.5% (*v*/*v*) Triton X-100 in PBS for 40 min at 4 °C, and washed two times in PBS. Samples were incubated in TUNEL reaction mixture (fluorescein isothiocyanate-conjugated dUTP and terminal deoxynucleotidyl transferase) at 37 °C for 1 h. TUNEL-stained blastocysts were washed with PBS and incubated in PBS containing 10 μg/mL Hoechst 33342 for 10 min. Blastocysts were washed and mounted onto a glass slide. Negative controls were prepared by omission of terminal deoxynucleotidyl transferase in the reaction mixture, and at the same time positive controls were prepared by pretreatment with 1 mg/mL DNase I (Roche Diagnostics, Basel, Switzerland) in 5 μL of Tris-HCl buffer, 1 μL of DNase, and 30 μL of H_2_O for 45 min at 37 °C. All samples were analyzed in a Nikon Eclipse 50i fluorescence microscope with filters for FITC (emission 520 and excitation 460–490) and Hoechst (emission 420 and excitation 330–385). TUNEL-positive blastomeres were fluorescently labeled green, indicating apoptosis. Blue (Hoechst) fluorescence indicated the presence of nuclei. Blastomeres were counted and analyzed using the open-source software Fiji/ImageJ. Apoptotic index was determined by dividing the total number of TUNEL positive blastomeres by the total number of blastomeres.

### 2.11. Gene Expression

Three replicates of expanded blastocysts treated with 0, 0.5% ethanol, 50, 100 and 200 µM α-Tocopherol were analyzed. All blastocysts were washed in Ca^2+^- and Mg^2+^-free PBS, and placed in 20 µL of RNA later™ Solution (Invitrogen, Waltham, MA, USA; Thermo Fisher Scientific, Waltham, MA, USA), snap-frozen in liquid nitrogen, and stored at −20 °C until gene expression analysis. Quick-RNA Microprep Kit (Zymo Research, California, USA) was used, for blastocyst RNA isolation followed by DNAse treatment using DNA-Free kit (Gibco), according to the manufacturer’s instructions. Total RNA concentration was determined by measuring the absorbance at 260 nm and its purity was evaluated at an absorption ratio of 260/280. For each sample, cDNA was synthesized by reverse transcription using a SuperScript III transcriptase (Thermo Fisher Scientific, Waltham, MA, USA) with random primers and 500 ng of total RNA as a template.

The expression of target genes was quantified using real-time PCR (qPCR). Primer sequences and the expected product lengths for genes *IFNT2*, *HSPA1A*, *CAT*, *SOD2*, *BCL2* and *BAX*, and the endogenous control *GAPDH* are presented in Table 1. The reactions used for real-time PCR were prepared using 7.5 μL of SYBRGreen master mix (Thermo Fisher Scientific, Waltham, MA, USA), equimolar quantities of the forward and reverse primers (10 µM), 2 µL of cDNA sample and 4.5 µL of RNAse/DNAse-free water at a final volume of 15 µL. Samples were amplified in duplicate in a Rotor-Gene 6000 72-disc rotor (Corbett Life Sciences, Sydney Australia). Standard amplification conditions were: 5 min at 95 °C and 40 cycles of 15 s at 95 °C, 40 s at 60 °C and 20 s at 72 °C. At the end of each run, melting curves were analyzed to ensure that the desired amplicon was detected, discarding contaminating DNA or primer dimers. A no-template control (NTC negative control) was used to corroborate the absence of contaminating DNA. The relative quantification of gene expression changes was recorded after normalizing for *GAPDH* gene expression computed using the 2^−∆∆CT^ method [30] in which CT value from a pool of all samples served as calibrator.

### 2.12. Statistical Analysis

Normality was checked with the Kolmogorov–Smirnov test, and homogeneity of variance was examined by Levene´s test. The Wilcoxon rank-sum test was performed for the data without normal distribution. Data were analyzed by one-way analysis of variance (ANOVA) with the Tukey test using SAS software (version 9.2; SAS Inst. Inc.; Cary, NC, USA). Results were expressed as the mean ± standard error of mean (s.e.m.). Differences between means were considered significant when *p* < 0.05.

## 3. Results

Table 2 shows environmental data recovered during the period of study. The average THI in the summer was around 72, indicating an alert level for cows under conditions of acute heat stress [31].

### 3.1. Experiment 1

**Figure 1:**
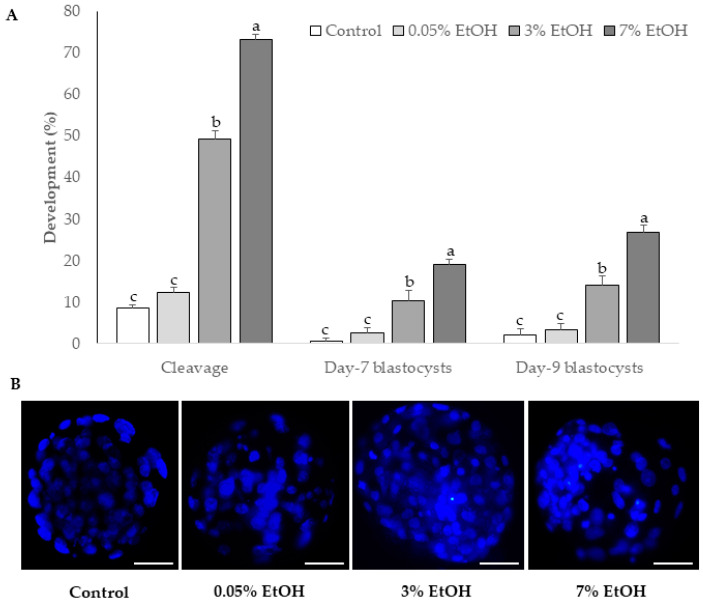
Effect of ethanol concentration on bovine oocyte parthenogenetic activation. (**A**) Percentage (% ± s.e.m) of cleavage (day 3), blastocysts’ development (day 7 and 9), and (**B**) blastomere nuclei stained with Hoechst in parthenogenic embryos derived from bovine oocytes matured without (control) or with 0.05% ethanol for 24 h or activated with 3% or 7% ethanol for 7 min. The white bar represents the 50 μm mark. Different letters above each bar indicate statistical significance (*p* < 0.05).

No significant differences were found in the proportion of mature (72.65 ± 0.65 and 72.14 ± 1.54; *p* = 0.77), immature (19.8 ± 2.7 and 19.82 ± 2.2; *p* = 0.9) and degenerate (7.56 ± 3.21 and 8 ± 2.28; *p* = 0.9) oocytes IVM for 24 h in presence (0.05%) or absence of ethanol, respectively. After 22 h of culture in absence of spermatozoa, oocytes from control and ethanol at 0.05% groups showed a clear delay in the formation of pronuclei (Table 3). Cleavage rate and parthenogenetic blastocysts rate were also significantly lower in both groups (Figure 1A). Representative digital images of parthenogenetic blastocysts stained with Hoechst 33324 are shown in Figure 1B. Parthenogenetic blastocysts total cell number did not differ significantly (*p* = 0.086) between control, 0.05%, 3 and 7% ethanol group (62 ± 3.0, 63 ± 2.67, 77.66 ± 5.58 and 75.77 ± 3.3, respectively).

### 3.2. Experiment 2

**Figure 2:**
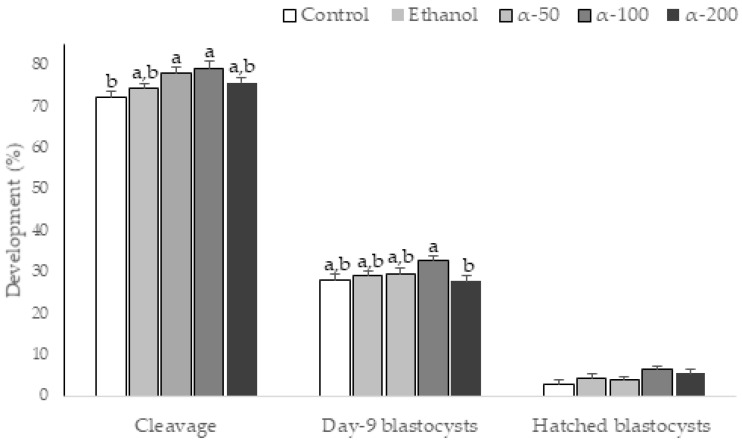
Effect of different α-tocopherol concentrations during bovine oocyte *in vitro* maturation on cleavage, blastocyst and hatching rates. Control: 0% ethanol and 0,0 α-tocopherol; Ethanol: 0.05% ethanol and 0,0 α-tocopherol; α-50: 50 µM α-tocopherol; α-100: 100 µM α-tocopherol; α-200: 200 µM α-tocopherol. Different letters above each bar represent significant difference (*p* < 0.05).

The percentage of oocytes reaching metaphase II and presumptive zygotes with normal fertilization were similar in all treatments (Table 4). The proportion of cleaved embryos derived from COCs supplemented with α-50 and α-100 was significantly higher than that in the control group (*p* < 0.05). The highest blastocysts rate on day 9 was obtained in the α-100 group, but it only differed significantly from the α-200 group. Hatched blastocyst rates were similar in all groups (Figure 2).

**Figure 3:**
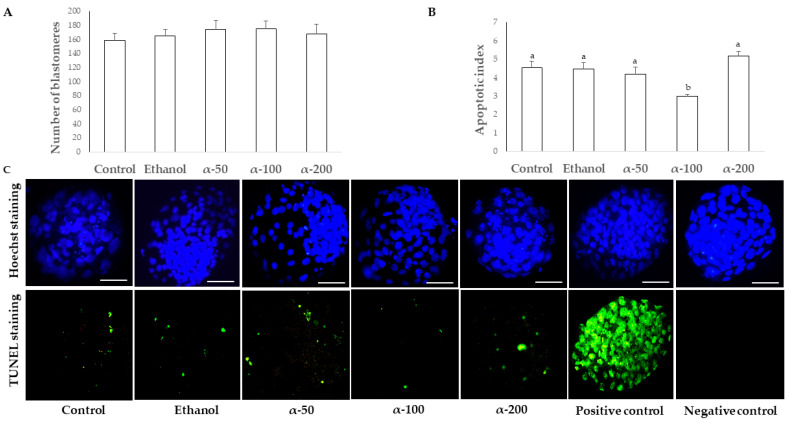
Effect of different α-tocopherol concentrations during bovine oocyte *in vitro* maturation on (**A**) total cell number, and (**B**) blastocyst apoptotic index. (**C**) Representative digital images of Hoechst and TUNEL staining of day 9 blastocysts derived from bovine oocytes collected during summer and *in vitro* matured in absence (control and 0.05% ethanol) or presence of α-tocopherol at 50 (α-50), 100 (α-100), 200 (α-200) µM, and positive and negative TUNEL assay controls. The white bar represents the 50 μm mark. Different letters above each bar represent significant differences (*p* < 0.05).

**Figure 4:**
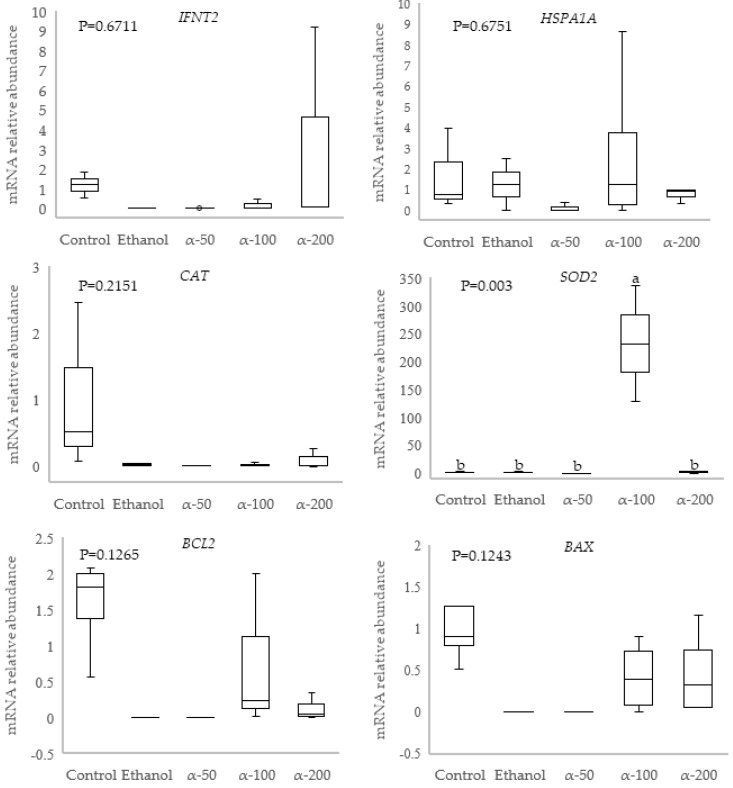
Effect of different concentrations of α-tocopherol during *in vitro* maturation of summer bovine oocytes on relative mRNA abundance of genes in expanded blastocysts of day 9 of *in vitro* culture. Interferon tau *(IFNT2)*; Heat shock protein 70 (*HSPA1A*)*;* Catalase (*CAT*); Manganese superoxide dismutase (*SOD2*); Bcl-2-associated X protein (*BAX*); B-cell lymphoma 2 (*BCL2*). Control: 0% ethanol and 0,0 α-tocopherol; Ethanol: 0.05% ethanol and 0,0 α-tocopherol; α-50: 50 µM α-tocopherol; α-100: 100 µM α-tocopherol; α-200: 200 µM α-tocopherol. Different letters above each box represent statistically significant difference (*p* < 0.05).

Although blastocyst total cell numbers did not differ among treatments (*p* = 0.825), however, the addition of 100 μM of α-tocopherol during IVM reduced the proportion of TUNEL-positive blastomeres (Figure 3B). A higher mRNA abundance was observed for *SOD2* (*p* < 0.05) in blastocysts in the α-100 group, compared to the other treatment groups, while no differences were detected in the expression levels of *IFNT2*, *HSPA1A*, *CAT*, *BCL2* and *BAX* (Figure 4) among groups.

## 4. Discussion

The results of the present study demonstrate for the first time that the use of ethanol at 0.05% (*v*/*v*) under 5% O_2_ during IVM does not promote parthenogenesis. The addition of α-tocopherol during IVM of summer-collected bovine oocytes affects blastocyst quality and yield in a dose-dependent manner. Supplementation of IVM medium with 100 μM α-tocopherol reduced the apoptotic index increased blastocysts quality and induced overexpression of *SOD2* in expanded blastocysts.

The delay in the proportion of pronuclear formation and low cleavage and blastocyst rates in both control and 0.05% ethanol groups indicate that our culture conditions do not promote bovine oocyte activation, meiotic resumption, or parthenogenetic development. In both groups, cleavage (8–12%) and blastocyst (1.5–2.8%) rates were similar to those reported by Méo et al. [32,33]. Spontaneous oocyte activation is influenced by prolonged culture times, 26 h for IVM and 44 h for IVM-IVF combined [23,34], and can indicate low oocyte quality or incomplete cytoplasmatic maturation [28]. Previous studies have demonstrated that summer-obtained oocytes, as was the case for oocytes in our study, are of lower quality than those collected during winter [9,10]. The levels of parthenogenetic activation in our control and ethanol groups could be attributed to spontaneous activation due to low oocyte quality.

Our results suggest that a single ethanol treatment (3 and 7% ethanol *v*/*v*) for a few minutes was effective for parthenogenetic activation. Recent studies have reported similar cleavage and blastocyst rates after bovine oocyte parthenogenetic activation with 5 µM ionomycin and 2 mM 6-dimethylaminopurine [35,36]. The combination of 5 µM ionomycin, 7% ethanol (*v*/*v*), and 50 µM roscovitine improved pronucleus formation rates in parthenogenetic embryos, while the highest blastocyst yields were observed in the ionomycin and 6-dimethylaminopurine treatment [24]. However, these protocols are long, involve several steps, and are difficult to reproduce. In addition, we isolated total RNA from parthenogenetic blastocysts, during preliminary experiments, and used it for successful qPCR standardization, indicating that parthenogenetic blastocysts had an active transcription machinery. Considering that artificial activation of bovine oocytes is a critical step for reproductive biotechnologies such as Intracytoplasmic Sperm Injection or Somatic Cell Nuclear Transfer [24], we propose this methodology for successful parthenogenetic activation of bovine oocytes. It could potentially be applied in other species as study material or stem cell source.

In the second experiment, the proportion of matured, normally fertilized oocytes and blastocyst cell numbers were not affected by the addition of α-tocopherol during IVM. Several studies show that the addition of α-tocopherol during maturation does not directly influence meiotic progression and IVF rate in cow [37], buffalo [38], sheep [39], pig [17,21,40], or rabbit [20]. Our results coincide with a previous study that did not observe any increase in total blastomere number in sheep blastocysts, after media supplementation with α-tocopherol during IVC [18]. Nevertheless, α-tocopherol improved the oocyte intrinsic competence, which can be assessed through embryonic development [20,41] as demonstrated in this study.

The addition of 100 µM α-tocopherol during IVM of bovine oocytes increased blastocyst rate, decreased apoptotic index and increased the expression of *SOD2* in 9-day blastocysts. These changes were not observed in groups supplemented with a lower or higher α-tocopherol concentration. However, the effects of this treatment were not associated with changes in the expression of *CAT, HSPA1A*, *BAX, BCL2* or *IFNT2*. SOD2 is a mitochondrial antioxidant enzyme that converts the anion superoxide (O^2−^) into H_2_O_2_ [42]. An overexpression of *SOD2* [43], but not of *CAT,* has been associated with higher embryo quality [44]. In fact, *SOD2* expression was downregulated in vitrified–thawed blastocysts [45] or blastocysts derived from heat-shock oocytes, which were cultured at 41 °C during IVM [22]. Recent studies revealed that an increase in *SOD2* expression, as shown in the present study, can be attributed to an effect of α-tocopherol on the expression of antioxidant genes [46].

The study conducted by Borcąri et al. [46] showed that diet α-tocopherol increased the expression of antioxidant genes and decreased the expression of prooxidant genes. α-tocopherol influenced epigenetic machinery and downregulated DNA methylation in specific genes in mice (revised by Chen et al. [47]). Our results suggest that the beneficial effect of α-tocopherol is dose-dependent, which agrees with a recent study showing that α-tocopherol exhibits positive effects on oxidative stress and epigenetic regulation involved in DNA repair in a dose-dependent manner [48]. In mice, vitamin E supplementation reduced DNA damage and affected *Dnmt1* and *MLH1* gene expression and methylation [49]. We could hypothesize that the addition of 100 µM α-tocopherol to the IVM medium improved the developmental potential and quality of blastocysts stimulating the expression of *SOD2*. However, the use of only two α-tocopherol concentrations in this study limited our understanding of the dose-dependent effect of α-tocopherol on oocyte maturation and competence.

In conclusion, 0.05% (*v*/*v*) ethanol does not induce parthenogenetic activation of bovine oocytes, therefore, at this concentration, ethanol can be used as a safe vehicle for hydrophobic molecules in embryo production systems. In contrast, a single >3% (*v*/*v*) ethanol treatment for 7 min is an effective procedure for parthenogenetic activation. The addition of 100 µM α-tocopherol diluted in 0.05% ethanol during IVM improves oocyte competence leading to an increase in blastocyst production and higher embryo quality, without the risk of inducing parthenogenetic activation.

## Figures and Tables

**Table 1:** Details of primers used for qPCR.

Gene Name	Gene Symbol	Primer Sequence (5′-3′)	Fragment Size (bp)	GenBank Accession No.
Interferon tau	*IFNT2*	F: TCTGAGGACCACATGCTAGG R: GATCCTTCTGGAGCTGGTTG	145	NM_001015511.3
Heat shock protein 70	*HSPA1A*	F: CTTCAACATGAAGAGCGCCG R: TGATGGGGTTACACACCTGC	182	NM_203322.3
Manganese superoxide dismutase	*SOD2*	F: CCCATGAAGCCTTTCTAATCCTG R: TTCAGAGGCGCTACTATTTCCTTC	307	NM_201527.2
Catalase	*CAT*	F: GTTCGCTTCTCCACTGTT R: GGCCATAGTCAGGATCTT	454	NM_001035386.2
Bcl-2-associated X protein	*BAX*	F: TTTGCTTCAGGGTTTCATCCA R: CCGATGCGCTTCAGACACT	126	NM_173894.1
B-cell lymphoma 2	*BCL2*	F: GAGTCGGATCGCAACTTGGA R: CTCTCGGCTGCTGCATTGT	120	NM_001077486.2
Glyceraldehyde 3-phosphate dehydrogenase	*GAPDH*	F: GATTGTCAGCAATGCCTCCT R: GGTCATAAGTCCCTCCACGA	94	NM_001034034.2

Abbreviations: F: forward; R: reverse.

**Table 2:** Maximum, minimum and average (±s.e.m) values for temperature (°C), relative humidity (%) and temperature-humidity index (THI) recovered between 21 December 2020 to 9 March 2021 reported in the north of Uruguay.

	Temperature (°C)	Relative Humidity (%)	THI
Maximal	25–29.8	87–94	75.78–83.94
Minimal	19.7–21.8	44–71	64.81–67.88
Average	23.31 ± 1.22	70.38 ± 5.05	71.31 ± 1.5

**Table 3:** Percentages (% ± s.e.m.) of pronucleus formation, telophase II, metaphase II + polar body, immature (germinal vesicle, metaphase I, anaphase I, telophase I) and degenerate in bovine oocytes cultured for 22 h (IVF-TL) in absence of spermatozoa and after of *in vitro* maturation without (control) or with ethanol at 0.05% for 24 h or activated with ethanol at 3% or 7% for 7 min.

	Control (% ± s.e.m.)	0.05% EtOH (% ± s.e.m.)	3% EtOH (% ± s.e.m.)	7% EtOH (% ± s.e.m.)	*p*-Value
Stages of nucleus					
2 Pronucleus	0	0	2.08 ± 2.0	5.00 ± 2.23	0.098
1 Pronuclei	7.91 ± 2.75 ^b^	7.69 ± 2.9 ^b^	19.37 ± 3.5 ^a^	25.09 ± 2.25 ^a^	0.006
Telophase II	4.16 ± 2.84	4.44 ± 2.93	9.12 ± 4.28	3.33 ± 3.0	0.62
Metaphase II + polar body	65.42 ± 2.00	58.73 ± 2.42	52.62 ±6.32	50.18 ± 3.68	0.062
Immature	10.27 ± 3.34	11.99 ± 3.91	4.16 ± 2.63	7.26 ± 2.32	0.32
Degenerated	12.21 ± 2.97	18.1 ± 1.07	15.96 ± 4.05	9.12 ± 3.42	0.21
Total oocytes evaluated	54	53	48	53	

Different letters in the rows indicate statistical significance (*p* < 0.05).

**Table 4:** Percentages (% ± s.e.m.) of mature (metaphase II, immature and degenerate) and fertilized (total, normal, polyspermic, asynchronous and not fertilized) bovine oocytes collected in summer and *in vitro* matured in absence (control and 0.05% ethanol) or presence of α-tocopherol at 50 (α-50), 100 (α-100) and 200 (α-200) µM.

	Control (% ± s.e.m.)	Ethanol (% ± s.e.m.)	α-50 (% ± s.e.m.)	α-100 (% ± s.e.m.)	α-200 (% ± s.e.m.)	*p*-Value
Nuclear maturation						
Metaphase II + polar body	71.66 ± 1.9	73.35 ± 3.17	71.7 ± 1.08	76.49 ± 1.89	76.54 ± 1.98	0.14
Immature	17.8 ± 2.32	20.28 ± 1.5	20.89 ± 2.74	16.66 ± 0.53	18.54 ± 2.01	0.63
Degenerated	9.58 ± 2.42	7.24 ± 2.4	7.46 ± 2.1	6.91 ± 1.96	5.08 ± 2.48	0.27
Total oocytes evaluated	73	69	67	66	59	
Fertilization rate						
Total fertilized	78.55 ± 1.81	75.89 ± 1.74	77.14 ± 2.79	80.58 ± 14	73.74 ± 2.77	0.25
Normal	60.1 ± 1.79	58.1 ± 2.26	59 ± 2.8	65.51 ± 1.71	60.12 ± 3.9	0.25
Polyspermic	5.92 ± 2.48	4.62 ± 2.34	5.27 ± 2	0	1.78 ± 1.78	0.2
Asyncronic	18.45 ± 1.76	17.78 ± 1.73	18.36 ± 1.58	15.06 ± 1.32	14.62 ± 3.86	0.5
Unfertilized	21.43 ± 1.81	24.09 ± 1.74	22.84 ± 2.79	19.4 ± 1.4	26.24 ±2.77	0.26
Total oocytes evaluated	71	73	70	66	73	

## Data Availability

In the results section the study presents all data generated.
